# The Nociceptin/Orphanin FQ System Is Modulated in Patients Admitted to ICU with Sepsis and after Cardiopulmonary Bypass

**DOI:** 10.1371/journal.pone.0076682

**Published:** 2013-10-04

**Authors:** Jonathan P. Thompson, Alcira Serrano-Gomez, John McDonald, Nadia Ladak, Sarah Bowrey, David G. Lambert

**Affiliations:** Division of Anaesthesia, Critical Care and Pain Management, Department of Cardiovascular Sciences, University of Leicester, Leicester Royal Infirmary, Leicester, United Kingdom; UNIFESP Federal University of São Paulo, Brazil

## Abstract

**Background And Objectives:**

Nociceptin/Orphanin FQ (N/OFQ) is a non-classical endogenous opioid peptide that modulates immune function *in* *vitro*. Its importance in inflammation and human sepsis is unknown. The objectives of this study were to determine the relationship between N/OFQ, transcripts for its precursor (pre-pro-N/OFQ [ppNOC]) and receptor (NOP), inflammatory markers and clinical outcomes in patients undergoing cardiopulmonary bypass and with sepsis.

**Methods:**

A prospective observational cohort study of 82 patients admitted to Intensive Care (ICU) with sepsis and 40 patients undergoing cardiac surgery under cardiopulmonary bypass (as a model of systemic inflammation). Sixty three healthy volunteers, matched by age and sex to the patients with sepsis were also studied. Clinical and laboratory details were recorded. Polymorph ppNOC and NOP receptor mRNA were determined using quantitative PCR. Plasma N/OFQ was determined using ELISA and cytokines (TNF- α, IL-8, IL-10) measured using radioimmunoassay. Data from patients undergoing cardiac surgery were recorded before, 3 and 24 hours after cardiopulmonary bypass. ICU patients with sepsis were assessed on Days 1 and 2 of ICU admission, and after clinical recovery.

**Main Results:**

Plasma N/OFQ concentrations increased (p<0.0001) on Days 1 and 2 of ICU admission with sepsis compared to matched recovery samples. Polymorph ppNOC (p= 0.019) and NOP mRNA (p<0.0001) decreased compared to healthy volunteers. TNF-α, IL-8 and IL-10 concentrations increased on Day 1 compared to matched recovery samples and volunteers (p<0.0001). Similar changes (increased plasma N/OFQ, [p=0.0058], decreased ppNOC [p<0.0001], increased IL-8 and IL-10 concentrations [both p<0.0001]) occurred after cardiac surgery but these were comparatively lower and of shorter duration.

**Conclusions:**

The N/OFQ system is modulated in ICU patients with sepsis with similar but reduced changes after cardiac surgery under cardiopulmonary bypass. Further studies are required to clarify the role of the N/OFQ system in inflammation and sepsis, and the mechanisms involved.

##  INTRODUCTION

 Sepsis remains a leading cause of admission to Intensive Care Units [1-3], with high mortality, costs, and long-term morbidity in those who survive [4]. The incidence of sepsis is estimated between 90-300 per 100,000 population [5,6] and the incidence of severe sepsis appears to have increased over the last decade [7-10]. Hence despite improvements in care, overall mortality has not reduced [8]: mortality from severe sepsis remains over 30% and higher in some groups and countries, which may reflect case mix, diagnostic criteria and availability of ICU resources [11-14]. Moreover, despite improvements in understanding of its pathophysiology effective new therapies for sepsis are lacking [15-18].

The clinical features of sepsis may be very similar to other non-infective inflammatory processes causing the systemic inflammatory response syndrome (SIRS) (major burns, acute pancreatitis) with similar consequences including multi-organ failure [18-20]. Cardiopulmonary bypass causes cytokine release including interleukins IL-1β, IL-6, IL-8, IL-18 and tumour necrosis factor-alpha (TNF-α) from activated endothelial cells. These stimulate the activation of several inflammatory pathways to promote systemic inflammation and organ dysfunction [19,20]. Higher cytokine concentrations are associated with worse outcomes after cardiac surgery [21,22]. Because of the similarities in inflammatory pathways involved in sepsis, SIRS and cardiopulmonary bypass, the latter can be used as a model of inflammation; better understanding may help differentiate between infective and non-infective systemic inflammatory responses [23].

Interactions between opioids and immune cells were demonstrated in 1909 [24] but more recent evidence suggests that opioids may modulate immune function directly [25-32]. Nociceptin/Orphanin FQ (N/OFQ) is an endogenous 17 amino-acid peptide that acts at the non-classical Opioid Nociceptin/Orphanin FQ receptor, NOP. The NOP receptor, along with messenger RNA (mRNA) for the N/OFQ precursor, pre-pro-N/OFQ (ppNOC), is widely expressed in the nervous system and other tissues including monocytes, lymphocytes and polymorphonuclear cells (PMNs) [33-35]. Several *in vitro* studies have shown that N/OFQ modulates immune function including chemotaxis and recruitment of human polymorphonuclear cells, lysozyme release from neutrophils, modulation of T cell function, inhibition of antibody formation, histamine release and increased cytokine release ( [36-41]; see 42 for review). In a rat caecal ligation-perforation model of sepsis, mortality increased (to 100%) after the parenteral administration of N/OFQ but was reduced in animals treated by a N/OFQ antagonist [43]. Stamer and colleagues reported reduced ppNOC mRNA expression in peripheral blood cells in critically ill patients with sepsis and increased NOP mRNA expression in non-survivors of sepsis, compared with healthy controls [44]. This accords with pilot data from our own group, showing that plasma N/OFQ concentrations were higher in non-survivors of sepsis [45].

Our hypothesis was that both NOP and ppNOC mRNA expression by polymorphonuclear cells, and plasma N/OFQ concentrations would increase during systemic inflammation and sepsis. The primary aim of this study was to test this hypothesis in patients admitted to Intensive Care with a clinical diagnosis of sepsis and in patients undergoing cardiac surgery using cardiopulmonary bypass (as a model of inflammation). Secondary aims were to analyze the relationship between changes in activity of the N/OFQ system with physiological and biochemical markers of inflammation and sepsis, and with clinical outcomes.

## Materials and Methods

### Patients with sepsis and matched volunteers

Eighty eight critically ill patients with a clinical diagnosis of sepsis admitted to the Leicester Royal Infirmary Intensive Care Unit were recruited between August 2009 and May 2011. The diagnosis of sepsis was made according to the attending physician on the basis of the presence of both infection and a systemic inflammatory response, in accordance with consensus criteria [46] (Table S1, Inclusion and exclusion criteria). Patients were not recruited if they were anticipated to die within 24 hours of ICU admission, or were taking part in another interventional study.

Blood samples were taken within 24 hours of admission to ICU or of developing sepsis (Day 1), 18-24 after the first sample (Day 2) and a further ‘recovery’ sample taken after the patient had made a clinical recovery from the episode of sepsis. Recovery samples were taken either in hospital or after discharge home. Day 1 and Day 2 samples were arterial blood drawn from an indwelling catheter and recovery samples were venous blood. Matched volunteer samples were taken from healthy individuals of the same sex and aged within 5 years of the patients with sepsis, with no evidence of systemic disease or current symptoms of infection (Table S1, Inclusion and exclusion criteria). Volunteers included healthy patients presenting for routine surgery, their relatives or members of staff. Venous samples were taken for full blood count, C-reactive protein, N/OFQ and cytokine measurement.

The clinical management of ICU patients with sepsis was at the discretion of the attending Intensivist and hospital protocols, but in all cases was consistent with the principles of the Surviving Sepsis Campaign [47]. This included source control, cardiovascular therapy guided by invasive monitoring, and other measures; antimicrobial therapy was guided by microbiologists according to the results of positive cultures where available. Patients were sedated with intravenous morphine and either midazolam or propofol, according to our Unit’s protocol at the time of the study.

### Patients undergoing cardiac surgery

Forty adult patients undergoing elective cardiac surgery with cardiopulmonary bypass (CPB) at Glenfield Hospital, Leicester, were recruited between July 2008 and January 2010. General anaesthesia and cardiovascular management were at the preference of the anaesthetist responsible for clinical care, but all patients received alfentanil 5–10 µg kg^-1^ or fentanyl 0.5–1mg, and propofol or etomidate until loss of eyelash reflex to induce anaesthesia. Maintenance of anaesthesia was provided using isoflurane 1–2% in an oxygen/air mixture and morphine 0.1–0.15mg kg^-1^ intravenously was administered at the end of surgery for analgesia. Arterial blood samples were taken before induction of anaesthesia (t0), 3 hours after the start of CPB (t3), and 18-24 hours after the start of CPB (t24).

### Ethics statement

This study includes two parts with separate ethics committee approval. It was approved by the North West London Research ethics committee 1 (ref: 09/H0722/21 for the study of ICU patients and healthy volunteers, and by the Leicestershire, Northamptonshire & Rutland Research Ethics Committee 1 (ref: 08/H0406/103) for the study of patients undergoing cardiac surgery). For ICU patients with sepsis, written consent or assent from the patient’s closest relative was obtained (prospectively where possible or retrospectively if not possible), with written consent in all cases from the patient when they had recovered. This process of retrospective assent followed by written patient consent after recovery was approved for this study by the Ethics Committee and the University Hospitals of Leicester NHS Trust Research & Development Department, as the study was non-interventional with no additional risks to the patient, and was time-sensitive. For healthy volunteers and cardiac surgical patients, prospective written informed consent was obtained.

### Blood handling: granulocyte preparation and extraction of plasma

A total of 22.5 ml whole blood was aspirated from the arterial catheter (in most of the cases where one was in place, otherwise blood was aspirated from the central venous catheter) or from a peripheral venous site in volunteers. Polymorphonuclear (PMN) leucocytes were isolated from 15mls of blood using Polymorphprep™ (Axis-Shield, Dundee, UK) following manufacturer’s instructions, within 30 minutes of the sample being taken. The polymorphonuclear band was harvested, washed with phosphate-buffered saline and then finally re-suspended in 1 ml of Tri-reagent® (Sigma-Aldrich, Dorset, UK) for RNA isolation. In some samples the granulocyte count was assessed by FACS as 98.4%, 99.7% and 99.8% for 1 volunteer and 2 patient samples respectively and these values corresponded to 83.8%, 85% and 94% respectively using a laboratory cell counter. In a total of 30 samples analysed using cell counting granulocytes ranged between 83.8% and 94%. Samples were stored at -80°C pending batch analysis. The remaining 7.5 ml blood was treated with aprotinin (0.6 TIU.ml^-1^) to prevent peptide/protein degradation transferred on ice to the laboratory and centrifuged and plasma separated by centrifugation. 1 ml aliquots were stored at -80°C until batch analysis of N/OFQ peptide by radioimmunoassay and cytokines by ELISA.

### Analysis for the Nociceptin/Orphanin FQ receptor (NOP) and pre-pro- N/OFQ (ppNOC) using quantitative Polymerase Chain Reaction (PCR)

Analysis of genetic material using quantitative PCR was performed using 2 housekeeper genes and in accordance with MIQE guidelines [48].

### RNA Extraction and Quantitative PCR

Isolated polymorphonuclear leukocytes (PMNs) were initially homogenized in cell lysis buffer, from a preparatory *mir*Vana RNA isolation kit (Applied Biosystems, Paisley, UK), and stored at -80°C. Total RNA was isolated from PMN homogenates using the *mir*Vana RNA isolation kit, which utilises a combination of organic and solid phase extraction methodologies. RNA samples were re-suspended in PCR grade water, RNA mass was determined using a NanoDrop (Labtech International, Uckfield, UK) and purity assessed from the 260/280nm ratio, typically >1.8. The integrity of extracted RNA was determined using an Agilent RNA 6000 Nano assay protocol (Agilent, Stockport, Cheshire, UK), RNA integrity numbers (RIN) were >9 in the cohort of samples tested.

For each sample a fixed mass of extracted RNA was processed using a Turbo DNA-*free* ^®^ kit, (Life Technologies Ltd, Paisley, UK) to remove genomic DNA (gDNA) contamination, in a limited number of samples RNA concentrations were too low to reach the set fixed mass, and the maximal amount possible for that sample was utilized. All samples were reverse transcribed using a High Capacity cDNA Reverse Transcription Kit (Labtech International, Uckfield, UK) according to the manufacturer’s instructions; copy DNA (cDNA) was then stored at -20°C.

cDNA samples were probed using quantitative PCR, suitable endogenous controls for the sample population were initially selected by screening against a series of common “housekeeper” genes, commercially available as TaqMan^®^ probes: human beta-glucuronidase (GUSB), human hypoxanthine-guanine phosphoribosyltransferaseHPRT1 (HGPRT), human TATA-box binding protein (TBP), Human beta-2-microglobulin (B2M) and human glyceraldehyde-3-phosphate dehydrogenase (GAPDH) (Applied Biosystems, Paisley, Scotland). Endogenous control data were processed using GeneNorm, (http://medgen.ugent.be/~jvdesomp/genorm/) with GAPDH and B2M suggested as the best panel of genes for normalizing those genes under investigation. cDNA samples were probed for transcripts that encode for the Nociceptin/Orphanin FQ receptor (NOP) and pre-pro- N/OFQ (ppNOC) using TaqMan gene expression assay (assay ID; Hs00173471_m1 and Hs00173823_m1 respectively). TaqMan probes for the genes under investigation (NOP, ppNOC) and the endogenous controls (GAPDH, B2M) were used with different fluorescent dyes, VIC and FAM respectively, allowing for Duplex measurements of both genes simultaneously. Reaction efficiencies for the Duplex assay format have been previously presented by our group and are within the range 90-110% [49]. Endogenous control data for GAPDH and B2M were normalized as geometric mean values.

The thermal profile for Q-PCR reactions in the StepOne instrument (Labtech International (Uckfield, UK) was 2min at 50°C, 10min at 95°C, 50 cycles of 15s at 95°C and 1min at 60°C. Non-template controls were included for all samples.

### Cytokine ELISA

Inflammatory cytokines (IL-8, IL-10, TNF-α) were measured in plasma obtained from whole blood as described above. Batch analysis of unextracted samples was performed using DuoSet® ELISA kits (R&D Systems, Abingdon, UK) according to the manufacturers’ instructions.

### Measurement of N/OFQ peptide concentrations: extraction and radio-immunoassay

N/OFQ solid phase peptide extraction was performed using Strata cartridges containing 200mg of C18-E and eluates of acetonitrile/TFA. Extracted samples were dried at room temperature using a centrifugal evaporator under vacuum and stored at -20°C until batch assay using a N/OFQ radioimmunoassay kit (Phoenix Europe Gmbh, Viktoriastrasse, Karlsruhe Germany) according to the manufacturer’s instructions and used previously by our group [45]. In this assay 38.2% of [^125^I] was bound and the IC50 for the standard curve was 27 pg ml^-1^, both within the manufacturer’s quality control reference values. Quadruplicate analysis of the positive control gave an intra-assay co-efficient of variation of 6.27%.

### Data analysis

Data were analyzed for normality distribution using D’Agostino and Pearson Omnibus, Kolmogorov-Smirnov and Shapiro Wilks tests using GraphPad Prism version 5.01 for Windows, (GraphPad Software, San Diego, California, www.graphpad.com). Most physiological data were non-normally distributed and so for clarity all are presented as median (interquartile range). Data were analyzed using Kruskal-Wallis Analysis of Variance (ANOVA) for unpaired data and Friedman’s ANOVA for paired data with Dunn’s multiple comparison test. P values <0.05 were considered statistically significant. In the absence of prior data, no formal power calculation was performed.

## Results

### Patients admitted to ICU with sepsis and matched volunteers

#### Patient characteristics

**Figure 1:**
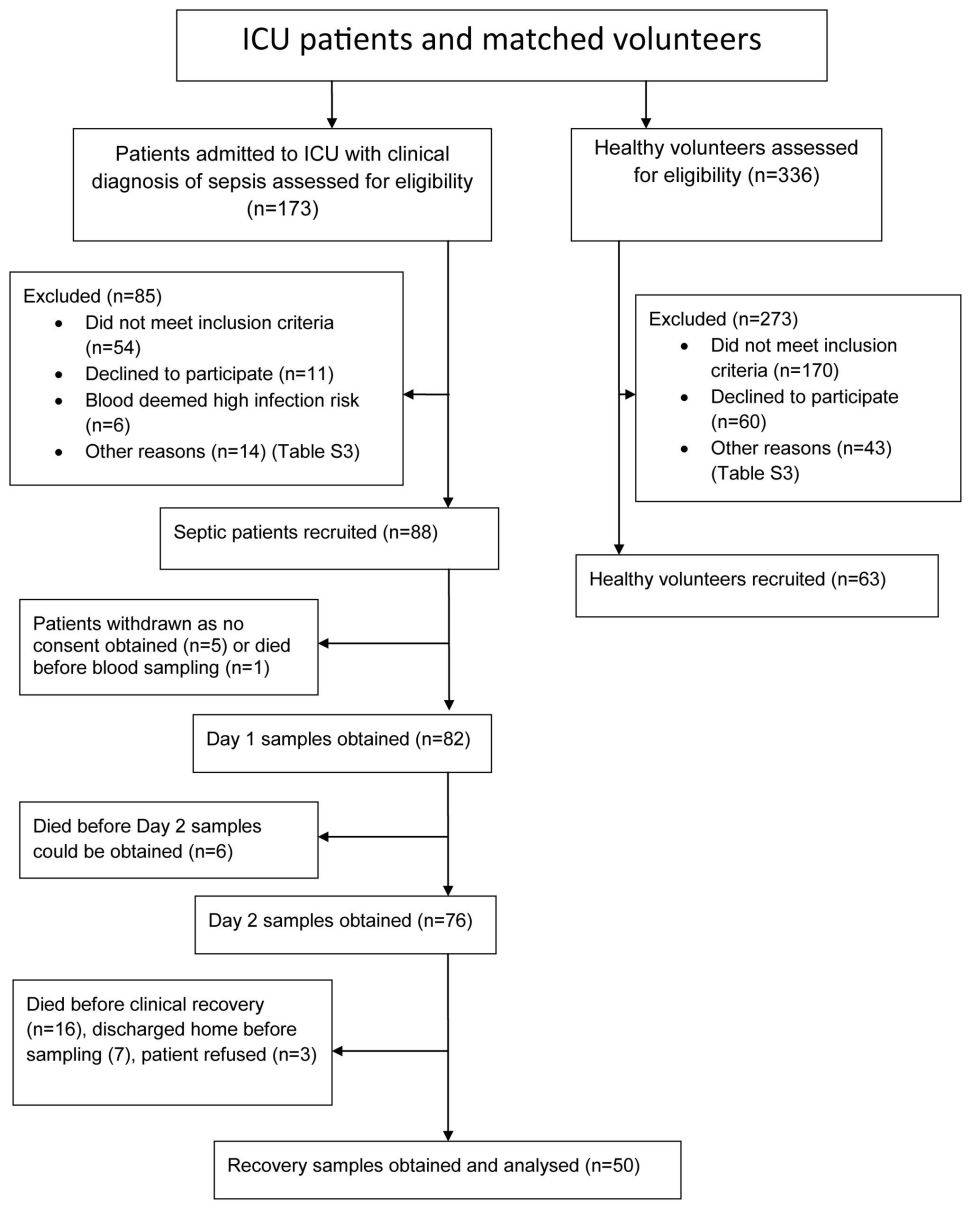
Study recruitment diagram: patients admitted to ICU with sepsis and matched volunteers.

**Figure 2:**
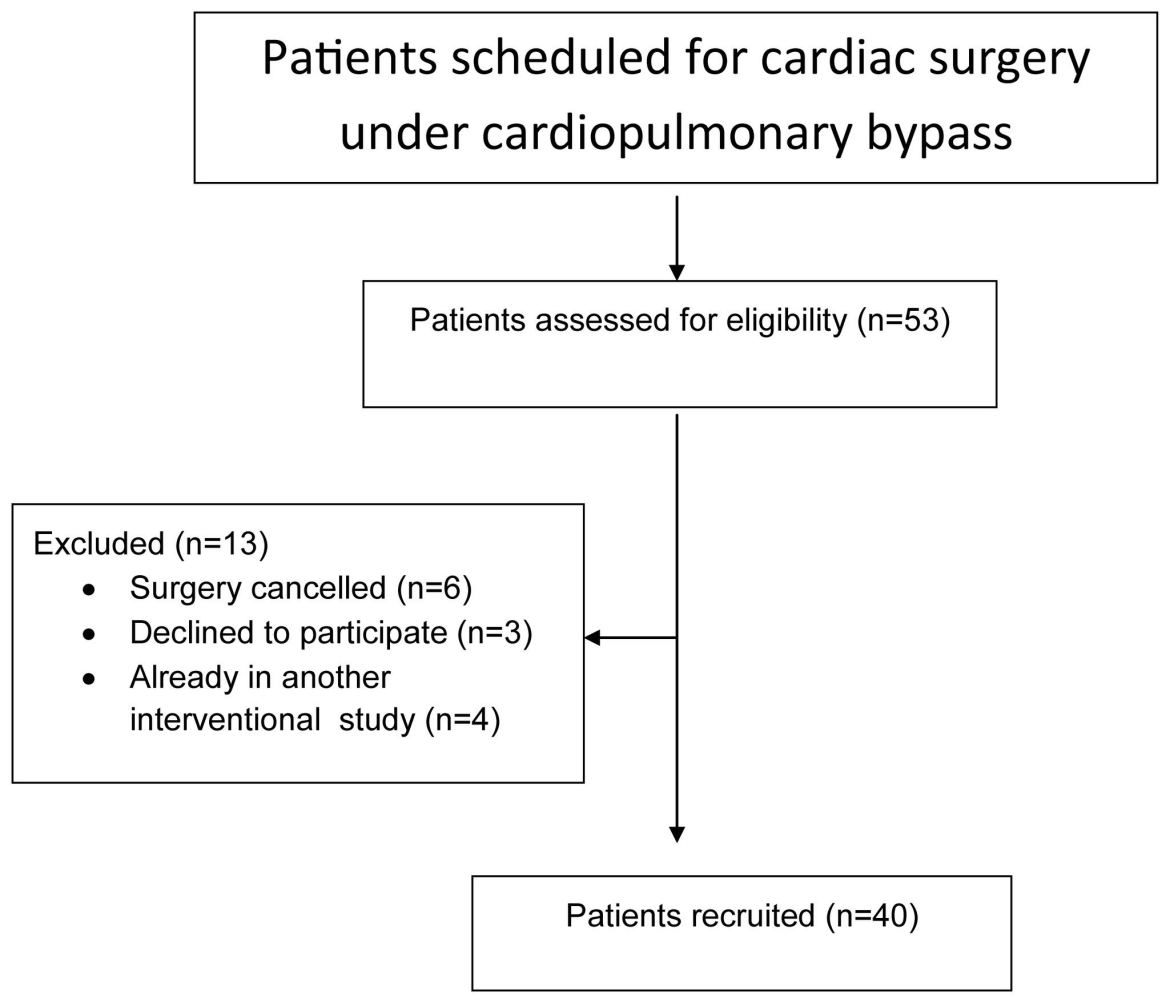
Study recruitment diagram: cardiac surgical patients.

One hundred and seventy three patients admitted to ICU with sepsis were screened (Figure 1). Of these, 85 were excluded because they did not meet the inclusion criteria (n=54), refusal to participate (n=11), their blood was considered a high infection risk for processing in our University laboratory (n=6), or other reasons (n=14) (Table S2). Day 1 samples were obtained from 88 patients but consent was later refused by 6 patients; Day 2 samples were obtained from 76 patients and recovery samples obtained from 50 patients (Figure 1). Patient characteristics, clinical and physiological data are detailed in Tables 1-2. Most patients were admitted with sepsis from a pulmonary or intra-abdominal source, and approximately half the patients were post-surgical. Causative organisms were isolated and confirmed in the majority of patients. Three hundred and thirty six healthy volunteers were screened to match according to age and sex of the ICU patients included in the study. Of the volunteers screened, 170 did not meet the inclusion criteria, 60 declined to participate, and 43 were excluded for other reasons (Table S2, Reasons for exclusion, healthy volunteers and patients with sepsis). Data from 63 healthy volunteers matched by age and sex to recruited ICU patients were therefore included in the main analysis (Figure 2, Table 2, Table S3, Characteristics of healthy volunteers).

**Table 1:** Patient characteristics in those admitted to ICU with sepsis (n=82).

Age (years)	62.5 (47-72)
Male/Female (n)	41/41
Weight (Kg)	73 (64-88)
BMI (kg m^-2^)	25 (23-29)
*Source of sepsis (n)*	
Pulmonary	41
Abdominal	26
Skin/soft tissues	5
Neutropenic- no source identified	5
Urogenital	3
Catheter-related	2
APACHE II score	20 (15-23)
SOFA score Day 1	7 (5-8)
Time from ICU admission with sepsis to enrolment (h)	15 (10-23)
ICU stay (days)	6 (3-11)
Hospital stay (days)	22 (11-41)
ICU mortality (n,%)	18 (22%)
Hospital mortality (n,%)	25 (30.5%)
30 day outcome (alive in hospital, discharged home, died)	29/31/22
Causative organisms*	
Gram negative	30
Gram positive	13
Anaerobes	2
Mixed organisms	5
Coagulase-negative staphylococci	5
Mycobacterium	1
Viral	1
None identified	25
Post-surgical	42
Time from ICU admission to recovery sample (d)	43 (19-71)
Ethnicity (White European, South Asian, Afro-Caribbean)	76/5/1
*Co-morbidities (n (%))*	
Cancer	21 (26%)
Hypertension	28 (34%)
Diabetes	13 (16%)
COPD/Asthma	11 (13%)
IHD	7 (9%)
Hypercholesterolaemia	7 (9%)
Chronic kidney disease	5 (6%)

COPD: chronic obstructive pulmonary disease; IHD: ischaemic heart disease

Data expressed as median (interquartile range) or number (%). * *Candida albicans* isolated from 8 patients but not considered the primary pathogen.

**Table 2:** Physiological and clinical variables in patients with sepsis on days 1 and 2 of admission to ICU.

**Physiological variable**	**Day 1 (n=82)**	**Day 2 (n=76)**	**Recovery (n = 50)**	**Healthy Volunteers (n = 63)**
Temperature (°C)	37.5 (36.9-38.3)	37.0 (36.6-37.8)	37.0 (36.5-37.0)	NC
MAP (mmHg)	78 (69-84)	78 (73-85)	85 (81-92)	95 (88-106)
HR (beats min^-1^)	107 (93-115)	99 (88-109)	95 (82-102)	NC
PaO2/FiO2 ratio	268 (138-354)	310 (186-376)	NC	NC
SOFA score	7 (5-8)	5 (3-8)	0 (0-1)	NA
Creatinine (µmol L^-1^)	117 (73-169)	106 (60-171)	55 (45-76)	NC
Hb (g L^-1^)	100 (89-114)	965 (890-1090)	109 (101-130)	142 (131-152)
WCC (x10^9^ L^-1^)	12.9 (5.1-19.4)	12.6 (7.3-20.7)	7.9 (6.2-10.5)	6.6 (5.3-7.7)
Neutrophils (%)	10.5 (3.5-15.5)	11.1 (6.1-17.7)	5.2 (3.9-7.6)	3.8 (3.1-5.2)
Platelets (x10^9^ L^-1^)	196 (101-334)	203 (118-292)	436 (266-555)	255 (213-291)
INR	1.5 (1.3-1.8)	1.3 (1.2-1.6)	NC	NC
ALT (IU L^-1^)	28 (19-56)	28 (18-50)	33.5 (8-128)	NC
APTT ratio	1.4 (1.3-1.7)	1.4 (1.3-1.7)	NC	NC
Bilirubin (µmol L^-1^)	10 (5-21)	9 (4-14)	6 (5-10)	NC
Albumin (g L^-1^)	21 (16-24)	21 (16-25)	33 (30-39)	NC
Arterial pH	7.32 (7.26-7.38)	7.35 (7.31-7.40)	NC	NC
Base deficit	-6.4 (-8.6--3.4)	-4.8 (-7.4--1.9)	NC	NC
Lactate (mmol L^-1^)	2.2 (1.2-3.4)	1.6 (1.1-2.6)	NC	NC
Noradrenaline (n)	56	38	NA	NA
Noradrenaline dose (µg kg^-1^ min^-1^)	0.21 (0.1-0.26)	0.20 (0.13-0.26)	NA	NA
C reactive protein (mg L^-1^)	205 (115-285)	223 (158-225)	29 (<5 - 60)	<5 (<5 - 18)
Adrenaline (n)	3	3	NA	NA
Requirement for renal support (CVVH)	10	11	0	0
Mode of ventilation				
Spontaneous/CPAP	26	22	50	63
NIV	1	0	0	0
MV	55	54	0	0

Data presented as median (interquartile range) or number.

NC = data not collected; NA = not applicable; CPAP = continuous positive pressure ventilation; NIV = non-invasive ventilation; MV = mechanical ventilation

#### Cytokine concentrations

Cytokine concentrations were increased on Days 1 & 2 of ICU admission in patients with sepsis (Table 3). Median values were highest on Day 1 compared to Day 2, recovery and volunteer samples respectively. The increases in IL-10 and TNF-α were statistically significant on Day 1 compared to recovery and volunteer samples (both p<0.0001), and for IL-8 the differences were significant at Days 1 and 2 (p<0.0001). There was a wide variation in cytokine concentrations, with several values at the lower limit of detection of the assays, but high values in other patients, reflecting the variability in clinical condition at the time of sampling. Cytokine concentrations in volunteers were low, as expected, with most values for IL-8, IL-10 and TNF-α being at the lower limit of assay detection. When data from just those volunteers who were matched by age and sex to patients with at least a Day 1 sample were compared, there were no major differences from analysis of the whole dataset (Table S4, Cytokine concentrations in patients admitted to ICU with sepsis on Days 1 and 2 of admission, and after clinical recovery from sepsis, and in a group of volunteers matched by age and sex to patients with sepsis). However early TNF-α concentrations were significantly higher, and there was a trend towards higher IL-8 values (p = 0.078) in patients who had died at 30 days compared to survivors (Table S5, Plasma cytokine and N/OFQ concentrations and mRNA expression for NOP and ppNOC, on Day 1 in patients admitted to ICU with sepsis, analysed according to 30-day mortality).

**Table 3:** Cytokine concentrations in patients admitted to ICU with sepsis on Days 1 and 2 of admission, and after clinical recovery from sepsis, and in a group of volunteers matched by age and sex to patients.

	**D1 (n=82)**	**D2 (n=76)**	**Recovery (n = 50)**	**Volunteers (n = 63)**
TNF-α (pg mL^-1^)	113 (50-189)*,†	85 (35-181)*	43 (16-160)	33 (17-78)
Interleukin 8 (pg mL^-1^)	277 (47-790)*,†	243 (41-563)*,†	34 (31-280)*	31 (31–31)
Interleukin 10 (pg mL^-1^)	187 (84-587)*,†	153 (55-430)*	47 (31-512)*	31 (31-220)

Data presented as median (interquartile range) and analyzed using Kruskall-Wallis ANOVA with Dunn’ post-test analysis. All cytokine concentrations were significantly increased compared with ‘recovery’ samples on Day 1 and in comparison with healthy volunteers. Concentrations remained significantly higher compared to volunteers on Day 2. Assay range for TNF-α was 16.6-1000pg mL^-1^ and for IL8 and IL10 was 31.3-2000pg mL^-1^. The lower limit of detection was set at the lowest standard value. Data at or below the lower limit of detection (LLD) were assigned the LLD value for analysis. In the healthy volunteers, 33 values were at the LLD for IL-8 and all 50 values were at the LLD for IL-10

* p<0.0001 compared to volunteer samples

† p<0.0001 compared to ‘Recovery’ samples

#### N/OFQ, ppNOC and NOP receptor mRNA expression

**Figure 3:**
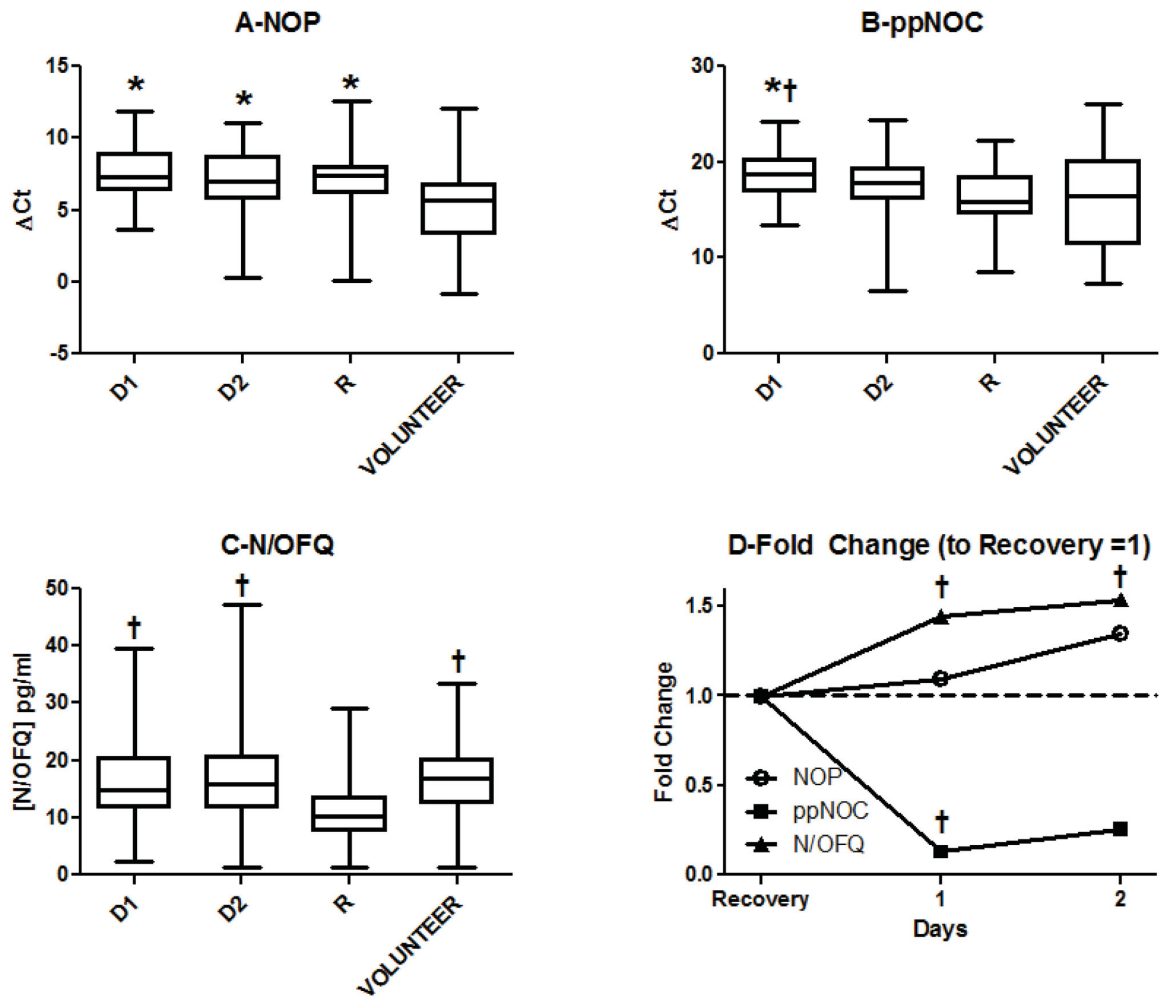
Effects of sepsis on NOP mRNA, (panel A) and ppNOC (panel B) mRNA, and N/OFQ (panel C) concentrations. Panel D represents fold change relative to recovery samples. Data are median, interquartile and full range and for PCR are presented as change in PCR cycle threshold relative to the geometric man of the two housekeepers used (ΔCT). Higher ΔCT values indicate more PCR cycles are required to detect the mRNA, and therefore less mRNA is being expressed. For NOP PCR samples on Day 1, in 1 sample there was no polymorph prep and one failed to amplify; for Day 2 samples there was no polymorph prep in 4 samples and 2 failed to amplify; for recovery samples there was no polymorph prep in 2 and 4 failed to amplify. For ppNOC PCR on Day 1, in one sample there was no polymorph prep and in 12 there was no amplification; for Day 2 samples there was no polymorph prep in 4 and no amplification in 10; for recovery data there was no polymorph prep in 4 and no amplification in 3; for the volunteer group 16 failed to amplify. Plasma N/OFQ measurements were made in all samples but in the volunteer group 3 samples were set at the limit of detection (1.25pg mL^-1^). Data were analyzed using Kruskal-Wallis analysis of variance followed by Dunn’s post-hoc testing; Figure 3 A-D: NOP (p<0.001) and ppNOC (p=0.019) mRNA values were lower in patients with sepsis when compared to volunteers and this was more pronounced during the first day in ICU (Figure 3, panels A and B). Plasma N/OFQ concentrations were higher on Days 1 & 2 of ICU admission compared to the recovery sample (p<0.0001, panels C and D). N/OFQ concentrations in recovery samples were reduced compared to volunteers (p<0.0001). *significantly different compared to volunteer; † significantly different compared to recovery samples.

Median (IQR) Day-1 ΔCt values for NOP and ppNOC in patients with a diagnosis of sepsis were 7.26 (6.41-8.95) (n=80) and 18.84 (17.00-20.24) (n=69) respectively (Figure 3). In volunteers these values were 5.70 (3.37-6.81) (n=61) and 16.48 (11.43-20.18) (n=47) respectively. NOP mRNA was 1758-3061 fold more abundant than ppNOC mRNA. NOP (p<0.001) and ppNOC (p=0.019) mRNA values were lower in patients with sepsis when compared to volunteers and this was more pronounced during the first day in ICU (Figure 3, panels A and B). Plasma N/OFQ concentrations were higher on Days 1 & 2 of ICU admission compared to recovery (p<0.0001). N/OFQ concentrations in recovery samples were reduced compared to volunteers (p<0.0001) (Figure 3, panel C). When data from just those volunteers who were matched by age and sex to patients with at least a Day 1 sample were analyzed, no further differences were found (Table S4). There were no significant differences in plasma N/OFQ or mRNA for NOP or ppNOC between 30-day survivors and non-survivors (Table S5). Similarly, when data were analyzed according to a diagnosis of cancer (n=17) or no cancer (n=65), there were no differences in cytokines, N/OFQ or mRNA (Table S6, Plasma cytokine and N/OFQ concentrations and mRNA expression for NOP and ppNOC on Day 1 in patients admitted to ICU with sepsis, analyzed according to whether they had a diagnosis of cancer).

### Patients undergoing cardiac surgery under cardiopulmonary bypass

#### Patient characteristics

Of 53 patients screened, 13 were ineligible because of cancelled surgery (n=6), refusal to participate (n=3) or involvement in another interventional study (n=4) (Figure 2). The characteristics of patients included are summarised in Table 4; full details are available as Supporting information (Table S7, Characteristics of patients undergoing cardiac surgery).

**Table 4:** Summary characteristics of patients undergoing surgery under cardiopulmonary bypass (CPB, n=40).

Age (years)	71 (62-76)
Male/Female (n)	28/12
BMI (Kg m^-2^)	28 (26-34)
Duration of CPB (mins)	89 (76-123)
Aortic cross clamp time	54 (25-74)
Surgical procedure (n (%))	
CABG	17
Valve replacement	15
CABG and Valve replacement	7
Aortic root replacement	1
ICU stay (days)	1 (1-4)
Hospital stay (days)	15.5 (10-32)
30 Day mortality (n (%))	1 (2.5%)

CPB: cardiopulmonary bypass; CABG: coronary artery bypass grafting

Data presented as median (interquartile range) or number (%). More detailed data are available in Table S1.

#### Cytokine concentrations

Interleukin-8 and Interleukin-10 concentrations increased significantly at 3 and 24 hours after the onset of cardiopulmonary bypass (both p<0.0001), though several values at 24 hours were at the lower limit of detection of the assay. TNF-α concentrations were increased only at 24 hours compared to preoperative levels (p=0.0158), confirming a moderate inflammatory response to cardiopulmonary bypass and cardiac surgery (Table 5).

**Table 5:** Cytokine concentrations in patients undergoing cardiac surgery under cardiopulmonary bypass at the start (t=0), 3 and 24 hours after the onset of CPB.

	t=0	t=3h	t=24h
TNF-α (pg mL^-1^)	55 (17-114)	61 (24-138)	66 (17-126)*
Interleukin 8 (pg mL^-1^)	31 (31-136)	91 (59-358)†	51 (31-378)†
Interleukin 10 (pg mL^-1^)	31 (31-141)	165 (81-434)†	82 (31-333)†

Data presented as median (interquartile range) and analyzed using Friedman’s ANOVA with Dunn’ post-test analysis. N= 40 at all time points. There were significant increases in Interleukin-8 and Interleukin-10 concentrations from 3 hours after the onset of CPB; the increase in TNF-α was significant at 24 hours. The assay range for TNF-α was 16.6-1000 pg ml^-1^, and for IL8 and IL10 was 31.3-2000 pg ml^-1^. Data at or below the lower limit of detection were assigned the LLD value for analysis.

* p=0.0158 compared to t=3

† p<0.0001 compared to t=0

#### N/OFQ, ppNOC and NOP receptor mRNA expression

**Figure 4:**
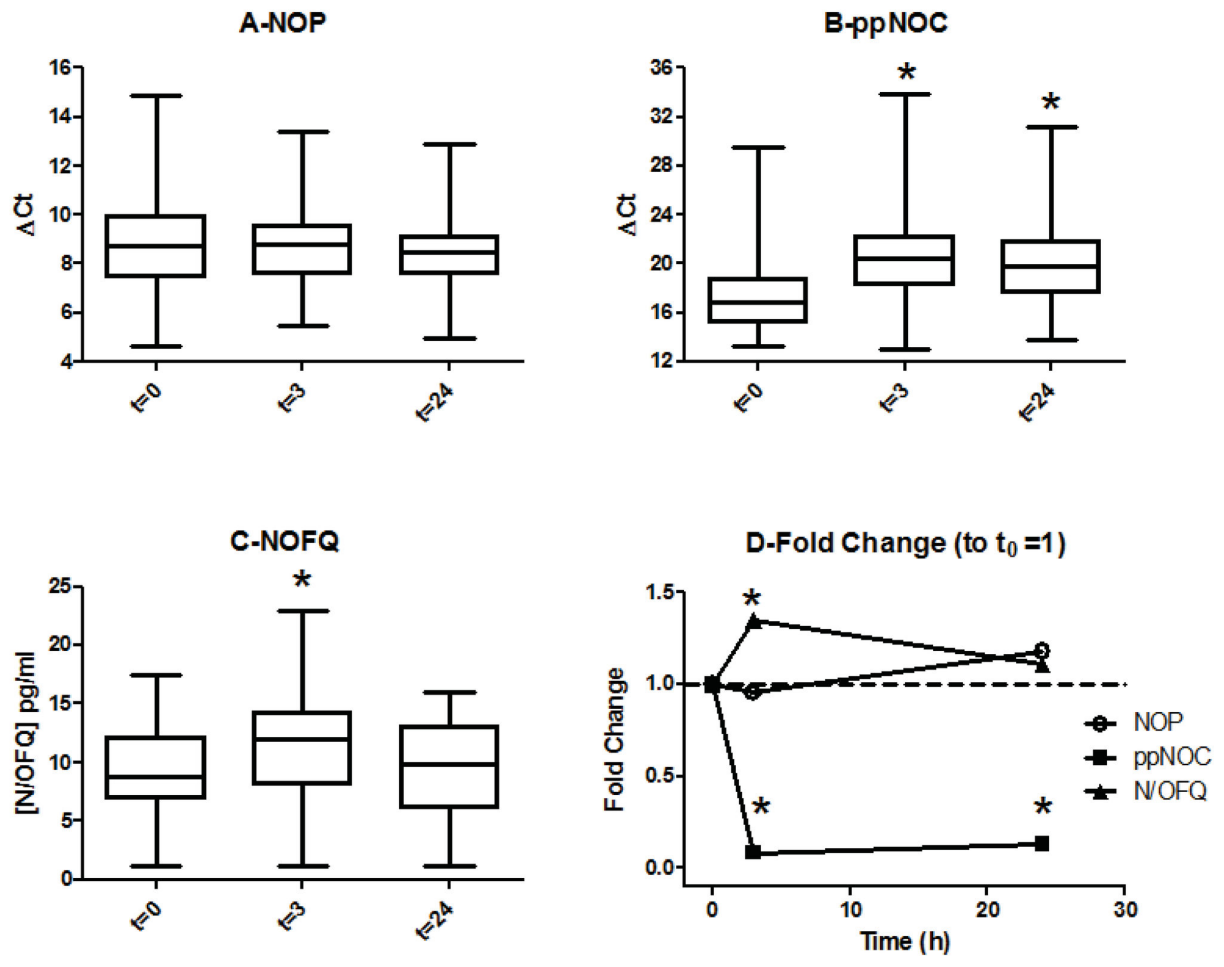
Effects of cardiopulmonary bypass on NOP (A) and ppNOC (B) mRNA, N/OFQ peptide (C) concentrations. Panel D represents fold change relative to pre bypass sample. Samples from 40 patients taken immediately before, (t=0), 3h (t=3) and 24h (t=24) hours after the start of cardiopulmonary bypass. A fold change summary is presented in D where it can be seen that following bypass there was a reduction in ppNOC mRNA and an associated increase in N/OFQ. Data presented as median, interquartile and full range and for PCR are presented as change in PCR cycle threshold relative to the geometric mean of the two housekeepers used (ΔCt). Higher ΔCt values indicate more PCR cycles are required to detect the mRNA, and therefore less mRNA is being expressed. For NOP, in 2 samples there was insufficient material for analysis at t=24h. For ppNOC PCR 6 samples at t=0, 4 samples at t=3h and 6 samples at t=24 failed to amplify and there was insufficient material for analysis of a further 2 samples at t=24. Plasma N/OFQ measurements were made in samples from all patients at all time points (n=120), but for 5 samples at t=0, 4 samples at t=3h and 3 samples at t=24h, measurements were set at the lower limit of detection (1.25pg mL^-1^). Data were analyzed using Kruskal-Wallis analysis of variance followed by Dunn’s post-hoc testing; Figure 4 A-C: There was no change in mRNA expression for the N/OFQ receptor NOP up to 24 hours after cardiac surgery (panels A andD). However, there was a significant decrease (increased ΔCt) in mRNA for the N/OFQ precursor, ppNOC at 3 hours after the onset of cardiopulmonary bypass, which persisted at 24 hours (p<0.0001, panels B and D). This was associated with a significant (35%) increase in N/OFQ at 3h (p=0.0058) that returned to basal values at 24h (panels C and D). *significantly increased compared to t=0. Figure 4D: * significantly different from t=0.

Median (IQR) t=0ΔCt values for NOP and ppNOC were 8.72 (7.48-9.95) (n=40) and 16.87 (15.19-18.83) (Figure 4). NOP mRNA was 210-471 fold more abundant than ppNOC mRNA. There was no change in mRNA expression for the N/OFQ receptor NOP up to 24 hours after cardiac surgery. However, there was a significant decrease (increased ΔCt) in mRNA for the N/OFQ precursor, ppNOC at 3 hours after the onset of cardiopulmonary bypass, which persisted at 24 hours (p<0.0001). This was associated with a significant (35%) increase in N/OFQ at 3h (p=0.0058) that returned to basal values at 24h.

## Discussion

In this study we found significant changes in the N/OFQ system in response to two different clinical states of systemic inflammation: cardiac surgery under cardiopulmonary bypass (inflammation without overt infection) and infection with inflammation (systemic sepsis). After cardiac surgery, plasma N/OFQ concentrations increased acutely, and expression of mRNA for ppNOC decreased, associated with moderate increases in the circulating pro- and anti-inflammatory cytokines, TNF-α, IL-8 and IL-10. Interleukin concentrations were decreasing 24 hours after the onset of cardiopulmonary bypass, consistent with other studies [19-22], in parallel with a reduction in plasma N/OFQ to baseline levels (Figure 4 panes C & D). Similar patterns were observed in ICU patients with sepsis: plasma N/OFQ concentrations increased during an episode of sepsis compared to recovery samples; mRNA for ppNOC decreased compared to recovery samples and matched volunteers; NOP mRNA decreased in patients with sepsis compared to volunteers, but there was no significant change between Day 1, Day 2 and recovery samples. More profound and significant increases in TNF-α, IL-8 and IL-10 were observed on the first 2 days of ICU stay, compared to samples taken after clinical recovery and in matched volunteers. These findings indicate that inflammatory processes stimulated acutely by cardiopulmonary bypass or over a longer period by severe sepsis produce significant changes in the N/OFQ system and that the changes are proportionately greater in response to sepsis. We attribute these proportionately greater changes in sepsis to the greater and ongoing inflammatory stimulus involved, compared to the limited and discrete stimulus of cardiac surgery; this is consistent with the time course of changes in N/OFQ and cytokine concentrations and the clinical characteristics of our patients. It is possible that there may be differences in the some of the cellular mechanisms underlying cytokine or N/OFQ system responses to sepsis or cardiopulmonary bypass, the effects of gender or gram positive/gram negative organisms [23] but this is uncertain and our study was not designed to address these possibilities.

It is increasingly apparent from laboratory and animal data that N/OFQ and NOP expression in immune cells enables immunomodulation, and has modulatory effects on the cardiovascular system [42], but there are limited data available on the effects of systemic inflammation *in vivo* on the N/OFQ system. N/OFQ has been shown to be released from human neutrophils in sites of inflammation [34] and to stimulate monocyte chemotaxis *in vitro* [37]. In a rat caecal-ligation model of sepsis, the administration of N/OFQ increased mortality, whereas the N/OFQ antagonist, UFP-101, reduced mortality and decreased TNF-α and other cytokines [43]. In an elegant study in humans, Stamer and colleagues reported significant reductions in peripheral blood cell ppNOC mRNA expression in 18 critically ill patients with sepsis and increased NOP mRNA expression in postoperative patients and non-survivors of sepsis, compared with healthy controls [44]. They also found similar patterns of increased NOP expression and decreased ppNOC in patients with terminal cancer, and an inverse relationship between procalcitonin concentrations and ppNOC mRNA expression, suggesting that the NOFQ system is perturbed in states associated with inflammation. However, there were no differences in NOP expression between survivors of sepsis and healthy controls. The reduction in ppNOC expression in sepsis is consistent with our data though we found no relationship between plasma N/OFQ or mRNA for NOP or ppNOC in patients with cancer or those without cancer. Other comparisons are limited because many of the patients in our study with a diagnosis of cancer had also undergone surgery, and none had terminal cancer. There may be differences in severity of illness, and in contrast to Stamer and colleagues, who used whole blood, we analyzed expression of ppNOC NOP mRNA by polymorphs; unlike Stamer’s study we also measured plasma N/OFQ peptide concentrations. However, taken together, these data suggest that polymorphs may not be the only source of ppNOC. The mechanism of reduced ppNOC expression might be increased consumption of ppNOC to produce N/OFQ, or reduced production because of negative feedback from high plasma concentrations of N/OFQ.

In a preliminary study, our group found increased plasma N/OFQ concentrations in critically ill ICU patients with sepsis who died compared to survivors [45]. Cytokine concentrations were higher in non-survivors, but in the present study we found no relationship between mortality and N/OFQ peptide concentrations. It is difficult to make firm conclusions because death in ICU patients is related to many factors other than severity of sepsis, including the presence of co-existing diseases, the development of unrelated complications, or withdrawal of treatment on the basis of futility. Moreover, our previous study was limited by small numbers, with only 4 deaths. The lower plasma N/OFQ peptide concentrations at recovery compared to matched controls is intriguing; although cytokine concentrations had normalised in most patients, they remained increased in a proportion of patients despite a clinical recovery from sepsis and suggests continued modulation of the N/OFQ system as inflammatory processes develop and subside.

This is the largest series of NOP, ppNOC and N/OFQ data in healthy volunteers and patients with sepsis. In most cases, a clinical diagnosis of sepsis was confirmed by microbiological testing, and the clinical data (median APACHE score of 20, SOFA score of 7 on Day 1, and requirements for artificial ventilation and inotropic support in the majority of patients) in conjunction with cytokine measurements confirm the severity of illness; this is also reflected in the ICU mortality of 22%. However, consistent with previous clinical studies in sepsis, there was significant variability in the severity of illness both in clinical and laboratory measures. This reflects the problems of clinical research in sepsis, including the limitations of current clinical definitions for sepsis, lead times to diagnosis and ICU admission, patient heterogeneity and other factors [8,12,50]. The wide variation in receptor and peptide expression in healthy volunteers confirms the wide inter-individual variability in the N/OFQ system and may have masked differences in response to sepsis.

We recognise some limitations within these data. Data were collected prospectively and it was not possible to collect recovery samples from all patients. However, when the data from just those patients with a complete dataset and a matched volunteer were compared the results were essentially similar. In addition, recovery from sepsis was defined as the patient being systemically well with no clinical signs of infection, but in a small proportion, cytokine concentrations and other biochemical markers of inflammation were above normal values, suggesting an on-going inflammatory process, and confirming the difficulty of diagnosing complete recovery from sepsis in clinical practice. In common with other studies, patients with sepsis were clinically heterogeneous. Whilst this introduces variability into the data, we have avoided extensive subgroup analysis to avoid misleading conclusions based on relatively small numbers in some of the sub-groups. We originally took samples on Days 1 and 2 of ICU admission to evaluate possible responses to resuscitation, but there were no differences between Day 1 and Day 2 data, and a longer interval may have been appropriate. The changes in N/OFQ plasma concentrations were relatively low, but large changes in ppNOC mRNA expression were seen. However, we have demonstrated a clear association between up-regulation of the N/OFQ system both after surgery under cardiopulmonary bypass and systemic sepsis. Concentrations of N/OFQ in other tissues were not determined, and whilst N/OFQ is probably produced by immune cells, its effect site is uncertain. Hence changes in plasma concentrations may underestimate its effects in inflammation. In terms of the experimental protocol, assessment of NOP expression is based on NOP mRNA measurements as there are no reliable antibodies to use in Western blots or fluorescence-activated cell sorting FACS analysis but we were able to assess both N/OFQ peptide and its precursor mRNA, ppNOC.

The consequences of increased plasma N/OFQ and possible up-regulation of the N/OFQ system are intriguing. It is well documented that N/OFQ produces negative chronotropic and inotropic effects *in vivo* in laboratory animals, and is a vasodilator of both innervated and non-innervated vessels [40,51]. We were not able to investigate a relationship between N/OFQ and cardiovascular function as we did not assess cardiac function directly and patients were receiving vasoactive medications in varying doses. In addition, our study comprised relatively small cohorts of a mixed population of ICU patients and those undergoing cardiac surgery, and so we would not be expect to demonstrate such differences. N/OFQ is immunomodulatory, with immune cells themselves expressing NOP and releasing N/OFQ; NOP activation affects neutrophil and monocyte chemotaxis [36,37]. The source of N/OFQ in our patient group could be neuronal or immune cells: we have clearly demonstrated the presence of ppNOC mRNA in polymorphonuclear leukocytes. By this mechanism, immune cell migration in response to an inflammatory or infective stimulus could lead to degranulation and release of N/OFQ; this could hold the immune cell at the site of inflammation and affect local vascular permeability.

## Conclusions

These data show that the N/OFQ system is modulated in humans both in sepsis and in response to cardiac surgery under cardiopulmonary bypass. The changes are proportionately greater and of longer duration in sepsis. The mechanism of increased plasma N/OFQ concentrations may result from increased utilisation of and hence a reduction in ppNOC mRNA within immune cells. These results are consistent with the established effects of the N/OFQ system in animal models on immune function, particularly polymorphonuclear leukocytes. Further work is required to confirm these findings, to elucidate the mechanisms involved and to explore the potential for therapeutic modification of the N/OFQ system in inflammation and sepsis. 

## Supporting Information

Table S1 **Inclusion and exclusion criteria for patients with sepsis and healthy volunteers.**
(DOCX)

Table S2 **Reasons for exclusion, healthy volunteers and patients with sepsis.**
(DOCX)

Table S3 **Characteristics of healthy volunteers (n= 63) expressed as median (interquartile range) or number.**
(DOCX)

Table S4 **Cytokine concentrations in patients admitted to ICU with sepsis on Days 1 and 2 of admission, and after clinical recovery from sepsis, and in a group of volunteers matched by age and sex to patients with sepsis.**
(DOCX)

Table S5 **Plasma cytokine and N/OFQ concentrations and mRNA expression for NOP and ppNOC on Day 1 in patients admitted to ICU with sepsis, analysed according to 30-day mortality.**
(DOCX)

Table S6 **Plasma cytokine and N/OFQ concentrations and mRNA expression for NOP and ppNOC on Day 1 in patients admitted to ICU with sepsis, analyzed according to whether they had a diagnosis of cancer.**
(DOCX)

Table S7 **Characteristics of patients undergoing cardiac surgery (n = 40), presented as median (interquartile range) or number.**
(DOCX)

Text S1 (DOC)
