# Supplementary material for: The Nociceptin/Orphanin FQ System Is Modulated in Patients Admitted to ICU with Sepsis and after Cardiopulmonary Bypass
Source: PLoS One. 2013 Oct 4;8(10):e76682. doi: 10.1371/journal.pone.0076682 (PMC3790749; doi:10.1371/journal.pone.0076682)
Supplement: Table S1 — Inclusion and exclusion criteria for patients with sepsis and healthy volunteers. (DOCX) [file pone.0076682.s001.docx]

**Table S1. Inclusion and exclusion criteria for patients with sepsis and healthy volunteers**

| ***Inclusion criteria patients*** | Two or more Systemic Inflammatory Response Syndrome (SIRS) criteria: core temperature of ≤36°C or ≥38°C; heart rate ≥ 90 beats min^-1^; respiratory rate of ≥20 breaths min^-1^ (or hyperventilation indicated by either a PaCO2 < 4.3 kPa or mechanical ventilation for an acute process); white blood cell count of ≤ 4x10^9^ l^-1^ or of ≥12×10^9^ l^-1^ (or the presence of greater than 10% immature neutrophils (bands) |
| --- | --- |
|  | Expected to survive at least 24 h after ICU admission |
|  | Age > 18 years) |
|  | Able to give informed consent, or assent taken from a relative with retrospective informed consent from the patient when able |
|  | Known or presumed infection |
| ***Inclusion criteria -healthy volunteers*** | Age (within ± 5 years) and sex matched to existing recruited ICU patient with sepsis |
|  | Able to give informed consent |
| ***Exclusion criteria -healthy volunteers*** | Cancer: current diagnosis (suspected or confirmed) or treatment for cancer within 2 years |
|  | Liver disease: active diagnosis or investigation for suspected liver disease |
|  | Rheumatoid arthritis: diagnosis at any time |
|  | Renal disease: CKD stage 3 or greater |
|  | Hypertension: diagnosis or receiving treatment |
|  | Insulin dependent diabetes mellitus |
|  | Infection: any confirmed or suspected infective process on the day of screening |
|  | Drug therapy: receiving any non-steroidal anti-inflammatory drug within 2 weeks, regular use of any opioid (and no doses within 24 hours), current antibiotic therapy, oral or parenteral steroid therapy within 4 weeks |
