# Supplementary material for: The Nociceptin/Orphanin FQ System Is Modulated in Patients Admitted to ICU with Sepsis and after Cardiopulmonary Bypass
Source: PLoS One. 2013 Oct 4;8(10):e76682. doi: 10.1371/journal.pone.0076682 (PMC3790749; doi:10.1371/journal.pone.0076682)
Supplement: Table S2 — Reasons for exclusion, healthy volunteers and patients with sepsis. (DOCX) [file pone.0076682.s002.docx]

**Table S2. Reasons for exclusion, healthy volunteers and patients with sepsis**

| ***Healthy volunteers: “other” reasons not recruited (N=43):*** | Needle phobia – 8 |
| --- | --- |
|  | No age match – 12 |
|  | Unsuccessful venepuncture – 2 |
|  | Volunteer unwilling to wait or insufficient time for blood sampling – 15 |
|  | Did not attend appointment for sample – 5 |
|  | Communication problems (poor command of English) – 1 |
| ***ICU patients: “other” reasons not recruited (N=14)*** | Next of kin not available for assent – 3 |
|  | No arterial access – 3 |
|  | Transferred to another hospital – 2 |
|  | Planned treatment withdrawal –2 |
|  | Learning difficulties – 1 |
|  | Patient died before sample could be taken – 1 |
|  | Psychiatric problems so informed consent not possible –1 |
|  | Already in another interventional study – 1 |
