# Supplementary material for: The Nociceptin/Orphanin FQ System Is Modulated in Patients Admitted to ICU with Sepsis and after Cardiopulmonary Bypass
Source: PLoS One. 2013 Oct 4;8(10):e76682. doi: 10.1371/journal.pone.0076682 (PMC3790749; doi:10.1371/journal.pone.0076682)
Supplement: Table S3 — Characteristics of healthy volunteers (n= 63) expressed as median (interquartile range) or number. (DOCX) [file pone.0076682.s003.docx]

**Table S3. Characteristics of healthy volunteers (n= 63) expressed as median (interquartile range) or number.**

| Age (years) | 60 (46-68) |
| --- | --- |
| Male/Female (n) | 29/34 |
| Weight (kg) | 74.5 (63-86) |
| BMI (kg m^-2^) | 25.1 (22.1-28.8) |
| Ethnicity (White European, South Asian, Mixed Afro-Caribbean) | 58/2/2 |
| Haemoglobin (g.l^-1^) | 142 (131-152) |
| WCC (x10^9^.l^-1^) | 6.6 (5.3-17.7) |
| Neutrophils (%) | 3.8 (3.1-5.2) |
| Platelet count (x10^9^.l^-1^) | 255 (214-290) |
| C reactive protein (mg ml^-1^) | <5 (<5 – 16) |
