# Supplementary material for: The Nociceptin/Orphanin FQ System Is Modulated in Patients Admitted to ICU with Sepsis and after Cardiopulmonary Bypass
Source: PLoS One. 2013 Oct 4;8(10):e76682. doi: 10.1371/journal.pone.0076682 (PMC3790749; doi:10.1371/journal.pone.0076682)
Supplement: Table S4 — Cytokine concentrations in patients admitted to ICU with sepsis on Days 1 and 2 of admission, and after clinical recovery from sepsis, and in a group of volunteers matched by age and sex to patients with sepsis. (DOCX) [file pone.0076682.s004.docx]

**Table S4. Cytokine concentrations in patients admitted to ICU with sepsis on Days 1 and 2 of admission, and after clinical recovery from sepsis, and in a group of volunteers matched by age and sex to patients with sepsis.**

| **Measure** | **Day1** | **Day2** | **Recovery** | **Volunteer** |
| --- | --- | --- | --- | --- |
| A. Plasma markers(pg ml^-1^) |  |  |  |  |
| N= | 63 | 61 | 41 | 63 |
| TNFα | 112(53-172)***** | 83(42-177)* | 44(16-188) | 33(17-78) |
| IL8 | 275(37-725)*, † | 250(41-709)* | 47(31-320)* | 31(31-31) |
| IL10 | 187(85-517)*, † | 142(56-420)* | 46(31-625) | 31(31-220) |
| N/OFQ | 17(12-22) † | 18(12-22) † | 10(7-13) | 17(12-19) † |
| B. PCR analysis |  |  |  |  |
| N= | 60 | 55 | 35 | 60 |
| ΔCT for NOP | 7.6(6.8-9.2)* | 7.1(6.1-8.8)* | 7.6(6.3-8.0)* | 5.6(3.4-6.8) |
| N= | 39 | 38 | 26 | 42 |
| ΔCT for ppNOC | 19.2(16.5-20.5)*† | 17.9(16.4-19.4) | 16.3(14.1-18.9) | 16.0(12.0-20.2) |

* p<0.0001 compared to volunteers’ samples

† p=0.0119 compared to ‘Recovery’ samples

Data from patients without ‘matched volunteer’ samples were excluded from this analysis. Data presented as median (interquartile range) and analysed using Kruskall-Wallis ANOVA with Dunn’ post-test analysis. PCR data are presented as change in PCR cycle threshold relative to the geometric mean of the two housekeepers used (ΔCT). Higher ΔCT values indicate more PCR cycles are required to detect the mRNA, and therefore less mRNA is being expressed.

All cytokine concentrations were significantly increased on Day 1 & 2 compared with matched volunteers. Interleukin 8 and 10 concentrations were higher on Day 1 compared with those at clinical recovery. Interleukin 8 concentrations at clinical recovery remained higher compared to volunteer samples. Plasma N/OFQ concentrations were significantly higher during Days 1 & 2 of ICU admission compared to clinical recovery, but were not significantly different from matched volunteer samples. mRNA expression of NOP was significantly lower in patients with sepsis compared to volunteers at all time points. mRNA expression of ppNOC was significantly lower on Day 1 of the episode of sepsis compared to recovery samples and volunteers. Assay range for TNF-α was 16.6-1000pg ml^-1^, for IL-8 and IL-10 was 31.3-2000pgml^-1^ and for N/OFQ was 10-1280pg ml^-1^(samples were concentrated as required). The lower limit of detection was set at the lowest standard value. In PCR some samples were not collected for PCR or PMNs could not be extracted

The results of this analysis are similar to that presented in the main paper (Figure 3), in which all patients with sepsis were included.
