# Supplementary material for: The Nociceptin/Orphanin FQ System Is Modulated in Patients Admitted to ICU with Sepsis and after Cardiopulmonary Bypass
Source: PLoS One. 2013 Oct 4;8(10):e76682. doi: 10.1371/journal.pone.0076682 (PMC3790749; doi:10.1371/journal.pone.0076682)
Supplement: Table S5 — Plasma cytokine and N/OFQ concentrations and mRNA expression for NOP and ppNOC on Day 1 in patients admitted to ICU with sepsis, analysed according to 30-day mortality. (DOCX) [file pone.0076682.s005.docx]

**Table S5. Plasma cytokine and N/OFQ concentrations and mRNA expression for NOP and ppNOC on Day 1 in patients admitted to ICU with sepsis, analysed according to 30-day mortality.**

| **Measure** | **Alive (n=62)** | **Dead at 30 days (n=20)** |
| --- | --- | --- |
| A. Plasma markers(pg ml^-1^) |  |  |
| TNF-α | 88(16-168) | 133* (16-276) |
| IL-8 | 230(31-633) | 853(31-3009) |
| IL-10 | 173(31-448) | 581(31-1116) |
| N/OFQ | 16(5-22) | 13(2-19) |
| B. PCR analysis |  |  |
| ΔCT for NOP | 7.3(6.4-8.6) | 7.18(6.3-9.2) |
| ΔCT for ppNOC | 18.9(16.7-20.2) | 18.3(17.6-20.7) |

* P = 0.029 between groups

Data expressed as median (IQR). Higher ΔCT values indicate more PCR cycles are required to detect the mRNA, and therefore less mRNA is being expressed. TNF-α concentrations were higher and there was a trend towards higher IL-8 concentrations (p=0.078) in non-survivors, but there were no significant differences in plasma N/OFQ or mRNA for NOP or ppNOC between survivors and non-survivors
