# Supplementary material for: The Nociceptin/Orphanin FQ System Is Modulated in Patients Admitted to ICU with Sepsis and after Cardiopulmonary Bypass
Source: PLoS One. 2013 Oct 4;8(10):e76682. doi: 10.1371/journal.pone.0076682 (PMC3790749; doi:10.1371/journal.pone.0076682)
Supplement: Table S6 — Plasma cytokine and N/OFQ concentrations and mRNA expression for NOP and ppNOC on Day 1 in patients admitted to ICU with sepsis, analyzed according to whether they had a diagnosis of cancer. (DOCX) [file pone.0076682.s006.docx]

**Table S6.**

**Plasma cytokine and N/OFQ concentrations and mRNA expression for NOP and ppNOC on Day 1 in patients admitted to ICU with sepsis, analyzed according to whether they had a diagnosis of cancer.**

| **Measure** | **Cancer (n=17)** | **No cancer (n=65)** |
| --- | --- | --- |
| A. Plasma markers (pg ml^-1^) |  |  |
| TNF-α | 112 (36-184) | 113 (51.9-190.2) |
| IL-8 | 535 (119-1596) | 217 (31-698) |
| IL-10 | 95 (73-559) | 187 (89-647) |
| N/OFQ | 15 (8-21) | 15 (12-21) |
| B. PCR analysis |  |  |
| ΔCT for NOP | 8.2 (6.9-9.2) | 7.2 (6.2-8.2) |
| ΔCT for ppNOC | 18.9 (17.3-19.9) | 18.8 (16.9-20.3) |

Data expressed as median (IQR). Higher ΔCT values indicate more PCR cycles are required to detect the mRNA, and therefore less mRNA is being expressed. There were no significant differences between groups.
