# Supplementary material for: The Nociceptin/Orphanin FQ System Is Modulated in Patients Admitted to ICU with Sepsis and after Cardiopulmonary Bypass
Source: PLoS One. 2013 Oct 4;8(10):e76682. doi: 10.1371/journal.pone.0076682 (PMC3790749; doi:10.1371/journal.pone.0076682)
Supplement: Table S7 — Characteristics of patients undergoing cardiac surgery (n = 40), presented as median (interquartile range) or number. (DOCX) [file pone.0076682.s007.docx]

**Table S7. Characteristics of patients undergoing cardiac surgery (n = 40), presented as median (interquartile range) or number.**

| Age (years) | 71 (62-76) | |
| --- | --- | --- |
| Male/Female (n) | 28/12 | |
| BMI (Kg.m^-2^) | 28 (26-34) | |
| ASA physical status classification (n (%)) |  | |
| III | 33 | |
| IV | 7 | |
| ***NYHA functional class (n, (%))*** |  | |
| 1 | 19 | |
| 2 | 14 | |
| 3 | 7 | |
| ***Surgical procedure (n (%)):*** |  | |
| CABG | 17 | |
| Valve replacement | 15 | |
| CABG + Valve replacement | 7 | |
| Aortic root replacement | 1 | |
| LV Ejection fraction (n (%)): |  | |
| > 50% | 25 | |
| 30 – 50% | 12 | |
| <30% | 3 | |
| Duration of anaesthesia (start of anaesthesia to end of surgery) (mins) | 249 (224-282) | |
| Duration of CPB (mins) | 89 (76-123) | |
| Duration of surgery (mins) | 199 (173-219) | |
| Blood transfusion before t3 (n ) | 12 | |
| Blood transfusion before t24 (n ) | 19 | |
| ***Vasoactive drugs (n)*** | T3 | T24 |
| Minimal (dopamine ≤5mcg.kg^-1^.min^-1^) | 10 | 7 |
| Moderate (dopamine >5mcg.kg^-1^.min^-1^ and/or adrenaline/noradrenaline ≤0.1mcg.kg^-1^.min^-1^) | 20 | 9 |
| High (dopamine >15mcg.kg^-1^.min^-1^, and/or adrenaline/noradrenaline >0.1 mcg.kg^-1^.min^-1^) | 2 | 3 |
| Intra-aortic balloon pump at t24 (n) | 4 | |
| ICU stay (days) | 1 (1 – 4) | |
| Hospital stay (days) | 15.5 (10-32) | |
| 30 Day mortality (n ) | 1 | |

t3 = 3 hours after induction of anaesthesia; t24 = 24 hours after induction of anaesthesia
