# Supplementary material for: The Nociceptin/Orphanin FQ System Is Modulated in Patients Admitted to ICU with Sepsis and after Cardiopulmonary Bypass
Source: PLoS One. 2013 Oct 4;8(10):e76682. doi: 10.1371/journal.pone.0076682 (PMC3790749; doi:10.1371/journal.pone.0076682)
Supplement: Text S1 — (DOC) [file pone.0076682.s008.doc]

Supporting information to:

**The Nociceptin/Orphanin FQ system is modulated in Systemic Inflammation and Sepsis.**

Jonathan P Thompson, Alcira Serrano-Gomez, John McDonald, Nadia Ladak, Sarah Bowrey, David G Lambert.

This supporting information contains additional analyses and additional data Tables S1-7.

**Analysis of plasma N/OFQ, mRNA for ppNOC and NOP, and cytokines according to 30 day mortality or a diagnosis of cancer.**

Stamer and colleagues reported significant reductions in peripheral blood cell ppNOC mRNA expression in 18 critically ill patients with sepsis and increased NOP mRNA expression in postoperative patients and non-survivors of sepsis, compared with healthy controls [1]. They also found similar patterns of increased NOP expression and decreased ppNOC in patients with terminal cancer, and an inverse relationship between procalcitonin concentrations and ppNOC mRNA expression, suggesting that the NOFQ system is perturbed in states associated with inflammation. However, they found no differences in NOP expression between survivors of sepsis and healthy controls. Therefore we compared plasma N/OFQ concentrations and cytokines, and polymorphonuclear leukocyte mRNA for ppNOC and NOP in our cohort, according to 30-day mortality or a diagnosis of cancer. In these analyses, there were no significant differences in plasma N/OFQ or mRNA for NOP or ppNOC between survivors and non-survivors (Table S5). Similarly, when data were analyzed according to a diagnosis of cancer (n=17) or no cancer (n=65), there were no differences in cytokines, N/OFQ or mRNA (Table S6).

**Reference:**

1. Stamer UM, Book M, Comos C, Zhang L, Nauck F, Stüber F. Expression of the nociceptin precursor and nociceptin receptor is modulated in cancer and septic patients. *Br J Anaesth*. 2011;106:566-72
